# Precision Medicine Using Simultaneous Monitoring and Assessment with Imaging and Biomarkers to Manage Mechanical Ventilation in ARDS

**DOI:** 10.1007/s44231-023-00045-4

**Published:** 2023-07-20

**Authors:** Megan Abbott, Yuchong Li, Laurent Brochard, Haibo Zhang

**Affiliations:** 1 Keenan Research Centre for Biomedical Science, St. Michael’s Hospital, Unity Health Toronto, Toronto, ON Canada; 2 Department of Physiology, University of Toronto, Toronto, ON Canada; 3 The State Key Laboratory of Respiratory Disease, Guangzhou Institute of Respiratory Disease, The First Affiliated Hospital of Guangzhou Medical University, Guangzhou, China; 4 Interdepartmental Division of Critical Care Medicine, University of Toronto, Toronto, ON Canada; 5 Department of Anesthesiology and Pain Medicine, University of Toronto, Toronto, ON Canada

**Keywords:** ARDS, Mechanical ventilation, Precision medicine, Computed tomography, Electrical impedance tomography, Lung ultrasound, Biomarkers

## Abstract

Acute respiratory distress syndrome (ARDS) has a ~ 40% mortality rate with an increasing prevalence exacerbated by the COVID-19 pandemic. Mechanical ventilation is the primary means for life-saving support to buy time for lung healing in ARDS patients, however, it can also lead to ventilator-induced lung injury (VILI). Effective strategies to reduce or prevent VILI are necessary but are not currently delivered. Therefore, we aim at evaluating the current imaging technologies to visualize where pressure and volume being delivered to the lung during mechanical ventilation; and combining plasma biomarkers to guide management of mechanical ventilation. We searched PubMed and Medline using keywords and analyzed the literature, including both animal models and human studies, to examine the independent use of computed tomography (CT) to evaluate lung mechanics, electrical impedance tomography (EIT) to guide ventilation, ultrasound to monitor lung injury, and plasma biomarkers to indicate status of lung pathophysiology. This investigation has led to our proposal of the combination of imaging and biomarkers to precisely deliver mechanical ventilation to improve patient outcomes in ARDS.

## Introduction

### Complications of Mechanical Ventilation in ARDS

Acute respiratory distress syndrome (ARDS) has a complex and uncontrolled inflammatory response caused by multiple insults such as pneumonia [25], COVID-19 [50], sepsis [25], acid aspiration [25], inhalation injuries [25] and trauma [25]. An influx of fluid and protein into the interstitial spaces and alveoli leads to heterogenous diffuse lung damage, resulting in pulmonary edema and subsequent fibrosis [17]. The ARDS mortality rate is as high as 30–50% [43].

ARDS patients receive life-saving support with mechanical ventilation (MV). MV is a significant confounder to improving patient outcomes, yet it remains the most valuable treatment [32]. A complication from MV is ventilator-induced lung injury (VILI). VILI is acute lung injury that results from or is aggravated by MV treatment and has physiological and biochemical mechanisms that lead to adverse patient outcomes [6, 32].

There are several mechanisms of injury: atelectrauma which results from the cyclic opening and closing of alveoli in collapsed lung areas; volutrauma from high tidal volumes putting excessive stress on the lungs; barotrauma due to high transpulmonary pressures that overinflate the lungs; and biotrauma that is a combination of atelectrauma and volutrauma, which increases the inflammatory response through the activation of neutrophils, macrophages and alveolar epithelial cells, and releases biomarkers [6].

Clinical guidelines have been developed to try to reduce VILI: positive end-expiratory pressure (PEEP) to keep alveoli open to reduce atelectrauma; low-tidal volumes (4–8 mL/kg of predicted body weight) to minimize volutrauma; and plateau pressure < 30 cmH_2_O to avoid barotrauma [20]. However, these strategies require precise individualization to protect patients’ heterogeneous lung injury and individual responses to MV. Therefore, the accuracy and effectiveness of the current delivery of MV for treatment are limited.

## Approaches for the Evaluation of Lung Mechanics and Mechanical Ventilation

The Pressure–Volume (P–V) curve is used to diagnose and monitor ARDS. It shows the relationship between the stage and severity of acute lung injury, assesses the volume recruited by PEEP, and the slope represents the compliance of the respiratory system [7]. However, the information provided by the P–V curve is evaluated under static conditions and may not be representative of the dynamic changes during MV [7].

The classic tool for diagnosis is the X-Ray, but it is very limited. The 2-D images do not account for gravity on the pleural pressure gradient and the ventro-dorsal distribution of lesions, and the consolidation of the dorsal lung can be hidden by the ventral aspect [62]. There is also a lack of assessment of dynamic lung aeration and function, and agreement between readers is low [62].

Advances in technology have led to tools that better assess lung mechanics and the effect of volume and pressure on the lungs. Computed Tomography (CT) delivers X-Ray imaging in 3-D. It can quantitatively assess the gravitational influence of alveolar aeration compared to atelectasis and lung tissue weight [24, 62]. It can evaluate lung mechanics, show the recruitment of collapsed or consolidated areas, and provides the highest resolution available to visualize the lung parenchyma for injury assessment [15, 62].

Electrical Impedance Tomography (EIT) shows real-time MV and monitors individual lung dynamics [33]. Ventilation-perfusion mismatch (V/Q) occurs when non-ventilated alveoli are perfused with pulmonary capillaries (shunt) or ventilated alveoli do not have sufficient blood flow to pulmonary capillaries (dead space), which can be assessed by EIT [3, 33]. Ventilation and perfusion maps show changes in quadrants to guide MV.

Lung ultrasound (LUS) can confirm and monitor the evolution of ARDS at the bedside by assessing changes in aeration, tissue density and PEEP-induced recruitment [2]. It can determine the efficacy of treatment and the ultrasound waves can also penetrate injured lung areas leading to targeted delivery of therapeutics [35, 46].

These tools each contribute to evaluating important components of the delivery of mechanical ventilation. Therefore, there are limitations in applying CT, EIT and LUS individually during MV. The gold standard for visualizing lung injury is CT and thus it is the most accurate tool for this purpose. However, CT requires patient transport and exposure to radiation, compared to LUS, which does not have as high spatial resolution, but can be easily applied at the bedside for monitoring injury progression. Detailed structural changes can be viewed on CT in contrast to EIT which has low spatial resolution, although EIT compensates for CT in its ability to visualize detailed functional changes and allows for the titration of ventilation settings for personalization. EIT can be used at the bedside, but it is an expensive technology.

Overall, these new technologies allow physicians to visualize MV distribution, assess individual responses to MV, and monitor measurements that are currently not examined at the bedside. This review will evaluate the current application of CT, EIT, LUS and plasma biomarkers in the context of dynamic management of MV, aiming to provide future direction on the best use of these tools to improve clinical outcomes in ARDS with precise treatment.

**Fig. 1:**
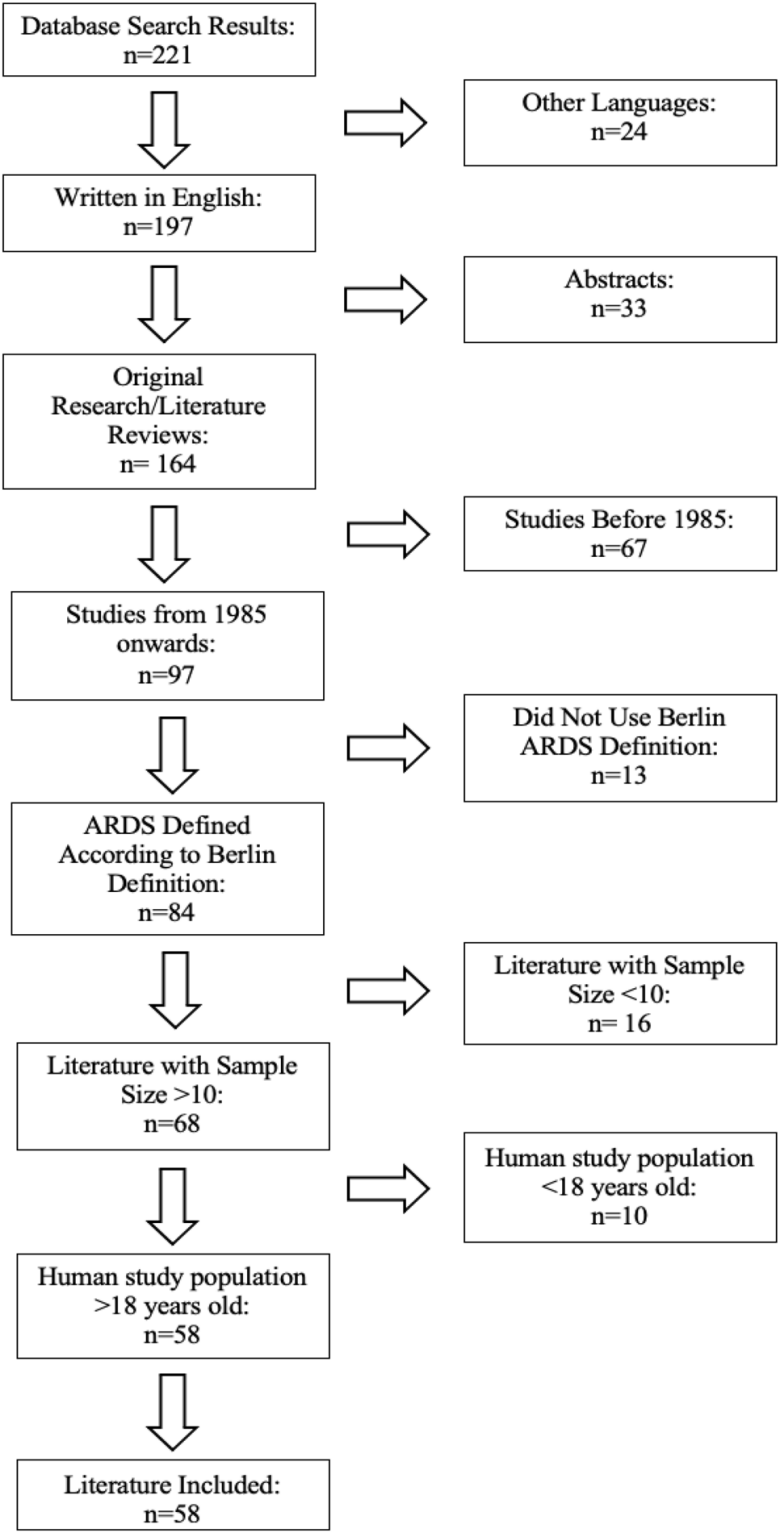
Identification of literature

An electronic database search of PubMed and Medline was conducted using keywords such as ARDS, VILI, LUS, CT, EIT, mechanical ventilation patient outcomes, ventilation perfusion, COVID-19, plasma biomarkers, delivery of therapeutics, animal models and human studies (Fig. 1).

## CT for Diagnosis and Evaluation of Lung Mechanics in ARDS and EIT to Monitor Lung Mechanics and Guide Ventilation

### Animal Models for CT

CT scans can quantitatively assess alveolar strain and lung tissue weight [41] as well as ventilation distribution in the lung [40]. Quantifying pulmonary fibrosis visualized on micro-CT using the Ashcroft scale (0–8) scored lung tissue with mild fibrotic changes between 0 to 3, and fibrotic masses and collagen from 4 to 5 [45]. The fibrosis measured by micro-CT correlated with the Ashcroft score, percentage of collagen content and alveolar air (r^2^ = 0.91, 0.77 and 0.94, respectively) [45]. The CT number categorized injury by a normal aerated range of − 1000 to − 500 or a poorly aerated range of − 500 to − 100 [54]. A shift in the CT number to − 300 correlated to the development of inflammation and fibrosis [31].

Mechanical power, defined as transpulmonary pressure, tidal volume and respiratory rate, was used to identify a VILI threshold. CT showed changes from isolated densities to whole-lung edema, indicating VILI above a threshold of 12 J/min [16]. The ratio between tidal volume, functional residual capacity and transpulmonary pressure determined a strain threshold. CT scans showed increased lung weight, stipulating VILI at a point between 1.5 and 2 [42].

### Human Studies for CT

The extent of the atelectatic lung on a CT scan classified patients with high and low recruitability with a sensitivity of 0.96, specificity of 0.76 and diagnostic accuracy of 0.86 [11]. Opacification in the anterior lung in 37.5% of patients represented fibrosis due to VILI [48]. Cross-sectional imaging identified opacification in 100% of ARDS patients, with consolidation in 75% of cases [48].

The percentage of alveolar fibrocytes was an independent predictor of mortality, associated with prolonged ventilation, and an indicator of poor prognosis in the early stages of ARDS [8]. Scoring pathology on a scale of 1 to 6, with a greater score indicating worse lung injury, showed that a CT score < 230 enabled survival prediction with a sensitivity of 73% and specificity of 75% [29]. Survivors had an overall smaller CT score (p = 0.002), a lower incidence of barotrauma (p = 0.013), and a greater number of ventilation-free days (p = 0.018) [29].

### Animal Models for EIT

There was an excellent correlation (r^2^ = 0.98) between the tidal volume and air delivered from the ventilator with < 6% mean error [30]. As PEEP was increased from 5 to 20 cmH_2_O, targeted re-aeration reached a lung volume of 87.1% of its normal value [30]. Air distribution was viewed with the estimated center of ventilation and anterior to posterior ventilation ratio. As PEEP was accurately titrated, the center of ventilation increased to 48.3%, almost reaching its normal value of 50%, anterior to posterior ratio was restored to 91% of its normal value, and the global inhomogeneity index decreased to its normal value [30].

EIT-tracked lung volumes maintained oxygenation, alveolar architecture, and lung mechanics better than low-tidal volume ventilation, while also decreasing histological evidence of VILI through a significant decrease in the presence of airway fibrin and hyaline membrane [49, 58]. PEEP determined by the global inhomogeneity index and hyperdistention indices led to the use of different FiO_2_ and PEEP combinations than the ones suggested by the ARDS network [28]. Identification of the onset of collapse and regional lung recruitment before changes in global pulmonary mechanics, occurred during PEEP titration [36].

### Human Studies for EIT

Individualization of PEEP and tidal volumes improved oxygenation (p = 0.0002) while reducing alveolar cycling without causing global overdistension (p = 0.0007, p = 0.015, stress and strain, respectively) [4]. Evaluation of early individualized PEEP in ARDS led to a 6% absolute reduction in mortality [27]. Guided PEEP titration was associated with higher weaning success, improved oxygenation and compliance, and an 18.3% increase in hospital survival in severe ARDS patients [61].

COVID-19 patients had a median value of 34% pixels with V/Q mismatch, with 6/7 patients having values > 30% [34]. The percentage of unmatched units in ARDS patients (% only ventilated units + % only perfused units) was significantly higher in non-survivors (p < 0.001) and an independent predictor of mortality (p = 0.004) with a sensitivity, specificity and negative predictive value of 77%, 87% and 91%, respectively [50].

## Assessment of Agreement between CT and EIT

### Animal Models

There were significant correlations (r = 0.98–0.99) between EIT and CT in assessing end-expiratory gas volumes, with a slightly lower correlation (r = 0.88) in tidal volumes [36]. Tidal recruitment on CT was strongly correlated to regional-ventilation-delay on EIT (r = 0.90–0.99, p < 0.05) [37]. Lung density measured by CT and EIT regional ventilation at increasing tidal volumes during PEEP titration had the strongest correlation in the dependent lung (r^2^ = 0.86) [22].

### Human Studies

In people with ARDS, when global CT-derived strain (Strain_CT_) was compared with simultaneous changes in electrical impedance (ΔZ) measured using EIT at end-inspiration and end-expiration, it revealed that ΔZ provides a real-time assessment of global cyclic strain at bedside, where the optimum PEEP with smallest lung strain is the PEEP where Δ*Z* is minimized, making EIT a potential surrogate for cyclic lung strain measured by CT (Strain_CT_) [12]. EIT regional ventilation changes in impedance showed a strong correlation (r^2^ = 0.92) to changes in lung density assessed by air content on CT [53]. Recruitable collapse estimated by EIT and CT showed good agreement at all levels of PEEP, with hyperinflation measured by CT and hyperdistention by EIT having good correlations (r^2^ = 0.85 and 0.95 in patients 1 and 2, respectively) [13]. A simulated EIT image with corrected lung areas based on CT, enhanced the global inhomogeneity index calculation, and increased sensitivity to alveolar collapse and overdistension [60]. EIT image reconstruction with CT showed that the ventilation distribution on EIT could be correlated to the underlying morphological information on CT [47].

## LUS for Diagnostics and Therapeutics

Management of patients on MV with LUS identified alveolar consolidation with a sensitivity, specificity and diagnostic accuracy of 100%, 78%, and 95%, respectively [59]. Classification of lung morphology (focal vs. non-focal) in ARDS during MV, using the Amsterdam method, had a specificity of 100% and sensitivity of 77% [39]. In patients receiving MV, the ventral LUS score was the most predictive of non-focal ARDS, with 100% specificity and 94% sensitivity [14]. The LUS total and ventral scores had an area under the ROC curve of 0.89 and 0.958, respectively [14]. Daily assessment of LUS scores indicated that a progressive reduction in the score was due to lung re-aeration and recovery, with a persistently high score corresponding to mortality [38].

Compared to normally aerated lung tissue that scatters LUS waves, injured lung areas can be penetrated by ultrasound due to the loss of aeration and fluid in the alveoli [46]. This allows for targeted delivery of therapeutics to injured lung areas [46]. Once LUS identifies the site of injury, therapeutics can be directly delivered to these areas, improving uptake, and reducing systemic effects [46].

## The Application of Plasma Biomarkers to MV

There are two lung epithelial biomarkers: Receptor for Advanced Glycation End-products and its soluble form (RAGE/sRAGE) and Surfactant Protein D (SP-D), which have been used for diagnosis and mortality in ARDS [55, 57]. Patients with high levels of RAGE benefitted from low-tidal volumes (p = 0.02), increases in SP-D indicated injurious ventilation and may be a marker of VILI (p = 0.02), with low tidal volumes attenuating these levels (0.0006) [10, 18, 19].

Angiopoietin-2 (Ang-2), a lung endothelial marker aided in the determination of ARDS severity and mortality, improved the ability of the Lung Injury Prediction score (area under-ROC = 0.84, p = 0.05) and augmented clinical scores by identifying patients who were developing or at high risk of lung injury during treatment [1, 21]. Interleukin-8 (IL-8) is a chemoattract immune cell and augmented diagnosis and morality in ARDS [9, 56]. IL-8 decreased during lung-protective ventilation (p < 0.05) [43].

A two-biomarker panel using Ang-2 and RAGE performed well across multiple patient cohorts of ARDS, with IL-8 and SP-D being the most frequently used biomarkers and having a higher predictive value in combination with clinical variables [55, 56]. Overall, these biomarkers indicate different aspects of the injurious and inflammatory mechanisms, and their combination improved clinical predictors [21, 23].

## Prospective

Using CT to visualize individual lung injury is important because understanding individual patient lung mechanics is linked to choosing ventilation strategies and patient outcomes. Most patients admitted to the Intensive Care Unit already have a CT scan, thus it is a feasible tool for this purpose. EIT is valuable for monitoring lung volumes and titrating ventilation settings to reach precisely determined values, but can also measure V/Q mismatch, which is not routinely assessed at the bedside. The current application of MV is not precise due to PEEP guidelines from the ARDS network not being adequate for all patients and with lung protective ventilation strategies being underused in ~ 30% of patients [5]. Therefore, there is a need for EIT to precisely guide ventilation according to validated indices, which would improve the current challenges of MV to potentially enhance patient outcomes.

CT and EIT are valuable tools independently, but also strongly correlate and have been validated against each other, with the structural capabilities of CT augmenting the functional aspects of EIT. Therefore, we propose their combination to precisely deliver MV by evaluating the lungs’ response to ventilation on EIT based on lung injury that can be visualized with CT. This approach considers the heterogeneous and individual lung responses in real-time, and the complexity of ARDS, but also provides clinicians with strategies that can accurately and effectively tailor MV compared to the current delivery. Although the studies used to inform this conclusion have not combined CT and EIT simultaneously, the analysis of their individual power and correlation between their measures shows their strong potential.

Regular monitoring of injury with LUS can be easily applied at the bedside to identify lung morphology in ARDS during MV. The loss of aeration in injured lungs also has utilization for therapeutics during MV. Advancements in using LUS at the bedside during MV are recent and the increase in patients during the COVID-19 pandemic led to its’ use to complement CT and monitor ARDS patients [52]. Treatment of MV includes using LUS to identify injury, followed by the precise delivery of therapeutics to the injured lung area. Therapeutics are currently not precisely distributed, leading to low treatment efficacy, which cannot be adequately evaluated due to a lack of routine injury assessment. Therefore, applying LUS to visualize injury and its’ progression, and for targeted delivery of therapeutics is another necessary component to precisely deliver treatment during MV.

Plasma biomarkers have prognostic value in ARDS and represent the underlying pathophysiology in the lung. They also augment clinical scores and MV strategies with statistical significance [1, 21, 55, 56]. We recommend the application of plasma biomarkers to elucidate the biochemical pathways in the lung that contribute to injury and patient outcomes during MV. Biomarkers could guide MV by indicating alterations in the lungs before they can be visualized. The biochemical contributors to VILI and ARDS are not currently assessed in the Intensive Care Unit, however, they are valuable because they could provide clinicians with a biological understanding of the efficacy of MV, which could lead to early changes in treatment.

## Conclusions

**Fig. 2:**
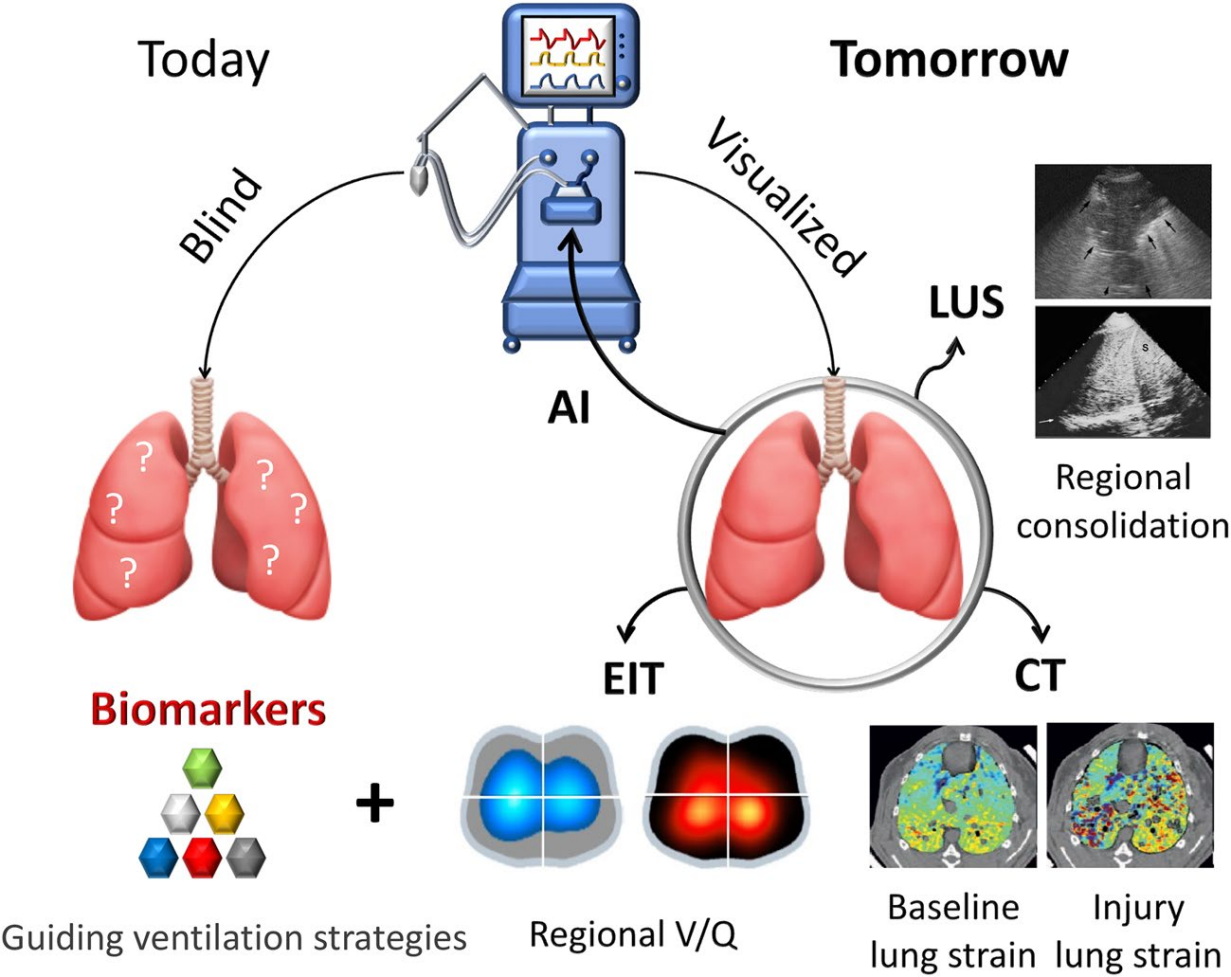
Proposed approach to manage mechanical ventilation (MV) in ARDS with precision medicine. The current MV approach is blinded regarding where pressure and volume is delivered into the lung by a ventilator. Systemic biomarkers maybe used as a result for evaluation of appropriate ventilatory strategies. The proposed approach is to use real-time bioimaging techniques, such as CT scan, electric impedance tomography (EIT) and lung ultrasound (LUS) to visualize and guide the pressure and volume delivered during MV in addition to biomarkers. EIT images are adopted [12], CT images [40] and LUC images (Galderisi 25)

Our evaluation of the literature assessing the utility of imaging and biochemical markers individually during MV has led to the proposal of their combination to provide precise management for ARDS patients (Fig. 2). Each imaging tool explored in this review provides vital information to guide and inform treatment with MV. Together, they have the potential to ameliorate the disadvantages of the current delivery of MV through precision medicine. A holistic understanding of the contributors to ARDS, VILI, and treatment during MV is imperative. Therefore, we have suggested the potential application of imaging and biomarkers to improve the delivery of MV, assess the progression of injury and to understand the pathophysiology in the lung. A summary of the value of each tool for key parameters are outlined (Table 1).

**Table 1:** Summary of key parameters assessed by CT, EIT and lung ultrasound

Technology	Parameters	Application to mechanical ventilation
Computed tomography	*Lung injury* Tissue density, fibrosis, edema and atelectasis *Lung mechanics* Recruitability, alveolar strain and aeration	1. Measures lung characteristics to target injured areas during MV 2. Evaluate changes in lung tissue as evidence of VILI 3. Assist with prognosis classification
Electrical impedance tomography	*Ventilation distribution* Center of ventilation ratio, anterior/posterior ratio, global inhomogeneity index and ventilation-perfusion match *Patient variation* Individualization of tidal volume and PEEP titration, visualization of lung recruitment and optimization of collapse vs. hyperdistention	1. Adjustments of ventilation settings to meet individual requirements for oxygenation, alveolar architecture and lung mechanics 2. Ability to decrease VILI through visualizing the distribution of ventilation 3. Optimal and personalized settings lead to improved survival and higher weaning success on MV
Lung ultrasound	*Lung injury* Alveolar consolidation and ARDS tissue patterns (focal vs. non-focal) with high sensitivity and specificity *Delivery of therapeutics* Non-aerated lung tissue can be penetrated by LUS for targeted delivery	1. Fast and reliable measures of the progression of lung injury during MV 2. Evidence to support shifts in MV settings based on changes in lung injury 3. Allows for targeted delivery of therapeutics to reduce systemic effects and improve uptake

An evaluation of the individual components of our proposed approach has been and continues to be tested to better understand the capability of each element. Further studies should be completed to test the simultaneous use of CT and EIT in animal models and then human studies, followed by the inclusion of plasma biomarkers to augment treatment decisions. LUS has strong evidence for identifying lung injury, but more research on its’ potential for delivering therapeutics in animals and then humans is necessary. Finally, these treatments should be combined and compared to the current delivery of MV to understand their full potential in improving patient outcomes in ARDS.

## Data Availability

Not applicable.

## Abbreviations

ARDS: Acute respiratory distress syndrome
CT: Computed tomography
EIT: Electrical impedance tomography
LUS: Lung ultrasound
MV: Mechanical ventilation
VILI: Ventilator-induced lung injury
PEEP: Positive end-expiratory pressure
V/Q mismatch: Ventilation perfusion mismatch
RAGE: Receptor for advanced glycation end-products
sRAGE: Soluble receptor for advanced glycation end-products
SP-D: Surfactant protein-D
Ang-2: Angiopoietin-2
IL-8: Interleukin-8
